# Genome-Wide DNA Methylation Enhances Stemness in the Mechanical Selection of Tumor-Repopulating Cells

**DOI:** 10.3389/fbioe.2020.00088

**Published:** 2020-03-17

**Authors:** Wei Huang, Hui Hu, Qiong Zhang, Ning Wang, Xiangliang Yang, An-Yuan Guo

**Affiliations:** ^1^National Engineering Research Center for Nanomedicine, College of Life Science and Technology, Huazhong University of Science and Technology, Wuhan, China; ^2^Center for Artificial Intelligence Biology, Hubei Bioinformatics & Molecular Imaging Key Laboratory, Key Laboratory of Molecular Biophysics of the Ministry of Education, College of Life Science and Technology, Huazhong University of Science and Technology, Wuhan, China; ^3^Department of Biomedical Engineering, College of Life Science and Technology, Huazhong University of Science and Technology, Wuhan, China; ^4^Department of Mechanical Science and Engineering, College of Engineering, University of Illinois at Urbana–Champaign, Urbana, IL, United States

**Keywords:** tumor-repopulating cells, DNA methylation, whole-genome bisulfite sequencing, dynamic changes, stemness

## Abstract

**Background:**

DNA methylation plays essential roles in tumor occurrence and stemness maintenance. Tumor-repopulating cells (TRCs) are cancer stem cell (CSC)-like cells with highly tumorigenic and self-renewing abilities, which were selected from tumor cells in soft three-dimensional (3D) fibrin gels.

**Methods:**

Here, we presented a genome-wide map of methylated cytosines for time-series samples in TRC selection, in a 3D culture using whole-genome bisulfite sequencing (WGBS).

**Results:**

A comparative analysis revealed that the methylation degrees of many differentially methylated genes (DMGs) were increased by the mechanical environment and changed from 2D rigid to 3D soft. DMGs were significantly enriched in stemness-related terms. In 1-day, TRCs had the highest non-CG methylation rate indicating its strong stemness. We found that genes with continuously increasing or decreasing methylation like *CREB5/ADAMTS6/LMX1A* may also affect the TRC screening process. Furthermore, results showed that stage-specific/common CSCs markers were biased toward changing their methylation in non-CG (CHG and CHH, where H corresponds to A, T, or C) methylation and enriched in gene body region.

**Conclusions:**

WGBS provides DNA methylome in TRC screening. It was confirmed that non-CG DNA methylation plays an important role in TRC selection, which indicates that it is more sensitive to mechanical microenvironments and affects TRCs by regulating the expression of stemness genes in tumor cells.

## Introduction

Cancer stem cells (CSCs) are high tumorigenicity (Najafi et al., 2019) and highly self-renewing (Hanahan and Weinberg, 2011) cells that play critical roles in tumor initiation and metastasis (Visvader and Lindeman, 2012). Our previous work has developed a mechanical method to select and/or generate CSC-like tumor-repopulating cells (TRCs) from cancer cells by culturing them in soft three-dimensional (3D) fibrin gels (Liu et al., 2012; Tan et al., 2014). TRCs selected from B16 mice melanoma cells were able to initiate tumors in wild-type mice with as few as 10 cells (Liu et al., 2012). Currently, studies have reported that 3D cell culture systems have contributed to tumor invasion/migration/metastasis, angiogenesis, microenvironment, and CSCs (Thiele et al., 2014). However, the regulation and mechanism on soft matrix mimic tumor niches contributing to tumor initiation and metastasis remain unclear. Thus, the process of TRC selection/transformation is a very good model to study the origin, regulation, and inner mechanisms of CSCs. Our previous work has provided insights into the mechanisms of TRC selection through transcriptome analysis (Huang et al., 2019). Currently, it is believed that DNA methylation plays an important role in maintaining the proliferation and differentiation of progenitor cells, as well as the stemness of CSCs (Sen et al., 2010).

DNA methylation can regulate gene expression and genome stability and plays a critical role in various processes, including embryo development (Jones and Baylin, 2002), cell growth and senescence, disease occurrence, tumor progression, etc. (Jones, 2012). DNA methylation in colorectal cancer (Draht et al., 2018), ovarian cancer (Baba et al., 2009), cervical cancer, liver cancer, and other cancer cells (Weisenberger, 2014) was reported to be closely related to gene expression changes in specific regions. Furthermore, many studies have shown that DNA methylation plays an extremely important role in CSCs. The difference of CSC marker gene *CD133* expression between CSCs and non-CSCs was related to DNA methylation (Yi et al., 2008). Tumor cells with significant characteristics of CSCs were observed in high expression of *OCT4*, *NANOG*, *SOX2*, *KLF4*, and other stemness genes, and co-upregulation of *Oct4* and *Nanog* could further increase the proportion of CSCs (Chiou et al., 2010). DNA methyltransferase (DNMT1) regulates DNA methylation and *de novo* synthesis enzyme, which is most critical for maintaining the characteristics of CSCs (Jones and Baylin, 2002). Proteins involved in the dynamic regulation of DNA methylation patterns are required for progenitor maintenance and self-renewal in mammalian somatic tissue (Sen et al., 2010). Therefore, DNA methylation is of great significance in CSC features and functions. Nonetheless, there is no study about a whole-genome DNA methylation dynamic change in CSCs origin.

In this report, we aim to study the whole-genome DNA methylation changes during the TRC selection process in soft matrix to learn more about the underlying epigenetic processes of the potential origin of CSCs (Nimmakayala et al., 2018). We sequenced HeLa 2D cells as a control and chose three sequential time points (1-day, 3-day, and 5-day) HeLa 3D to study the process of TRC selection and transformation. To address these issues, we compared the epigenetic status of 1-day versus 0 h (1-day/0 h), 3-day versus 1-day (3-day/1-day), and 5-day versus 3-day (5-day/3-day) to identify the differentially methylated regions (DMRs) and differentially methylated genes (DMGs). This comprehensive study on the dynamic changes of DNA methylation about the origin of TRCs may provide insight into the acquisition of cancer stemness and potential targets for tumor therapy.

## Materials and Methods

### Cell Lines and Cell Culture

Human cervical cancer cell line HeLa was maintained in our laboratory and was authenticated by the STR profiling by China Center for Type Culture Collection, Wuhan, China (CCTCC) in July 2017, and no cross-contaminated cell line was detected. HeLa cells were cultured on a plastic plate in Dulbecco’s modified Eagle’s medium (DMEM) (high glucose DMEM) with 10% fetal bovine serum (Life Technologies) and 100 mg/ml penicillin/streptomycin at 37°C with 5% CO_2_. The cells were randomly assigned to each experimental group, and the potential presence of mycoplasma was monitored via continuous microscopic imaging.

### HeLa 3D Preparation

HeLa cells were digested with trypsin (Life Technologies) from the 2D plastic plate, then were divided into single cell suspension. Fibrinogen was diluted into 2 mg/ml with T7 buffer (pH 7.4, 50 mM Tris, 150 mM NaCl). Cell plus fibrinogen mixture was made by mixing the same volume of single-cell solution and fibrinogen solution; the mixture’s concentration was 1 mg/ml fibrin gel (corresponding stiffness is 90 Pa). For the DNA extraction experiment, a 250-μl cell and fibrinogen mixture was seeded into each well of 24-well plate and mixed well with pre-added 5 μl thrombin (100 U/ml). The cell culture plate was then incubated in a 37°C cell culture incubator for 25 min. Finally, 1 ml DMEM with 10% fetal bovine serum and antibiotics were added.

### DNA Methylation Sequencing

HeLa cells cultured in the rigid plastic plate were marked as HeLa 2D (0-h sample described in the article), and in 90-Pa fibrin gels were marked as HeLa 3D. The cells, prior to being cultured into fibrin gels, were named as 0 h. HeLa 3D cells were collected in a row at three time points: 1-day (24 h), 3 days (72 h), and 5 days (120 h), which are the 1-, 3-, and 5-day samples mentioned in the article. The whole-genome DNA was extracted with an E.Z.N.A.^®^ MicroElute Genomic DNA Kit (Omega Bio-tek) following the instructions. Libraries for whole-genome bisulfite sequencing (WGBS) were prepared according to Illumina’s TruSeq protocol. The libraries were sequenced via platform (HiSeq X Ten) by BGI-Shenzhen (Wuhan, China). The sequencing data were processed using the Illumina analysis pipeline with a 2 × 100 bp paired-end strategy, and we obtained 6.67 × 10^7^ clean reads for each sample (Supplementary Table S1). Clean reads were then mapped to the reference genome (hg19) using the mapping software BSMAP (Xi and Li, 2009). Sequencing reads with low-quality bases (quality value ≤ 20 and the ratio of low-quality base >10%) were removed from the raw data prior to alignment of the sequence reads to the reference genome, using the analysis tool SOAPnuke, developed by BGI-Shenzhen^1^. The mapped rates of the whole genome varied from 82.18 to 84.48%, and mapping reads of CG (CpG), CHG, or CHH (mCHG and mCHH, where H corresponds to A, T, or C) were also shown (Supplementary Table S2). All downstream analyses were based on high-quality data.

### Methylation Level

The average DNA methylation level was calculated by the number of reads that support methylation divided by the total reads number that cover cytosine sites (Schultz et al., 2012). It was calculated as follows:

$${\text{Rm}}_{\text{average}}=\frac{{\text{Nm}}_{\text{all}}}{{\text{Nm}}_{\text{all}}+{\text{Nnm}}_{\text{all}}}\times 100\%$$

Rm_average_ represents the average level of methylation, Nm is the read number of methylcytosines (Petrova et al., 2014), and Nnm is the read number of unmethylated cytosine (non-mC). When calculating the levels of different methylation types in the whole genome, only the specific type of methylated cytosine (mC) was used. For example, RmCHG = NmCHG/(NmCHG + NnmCHG); here, only mC of CHG and non-mC of CHG were counted. For the methylation level of a particular region, the methylation rate of each region = (the total reads number that supported methylation in the region)/(the total reads number that supported methylation in the region + the total reads number that supported unmethylation in the region). Method of methylated cytosines identification and calculation was based on Lister et al.’s (2009) article, and the methylation level of C in the context of CG/CHG/CHH was calculated per strand.

### Detection of Differentially Methylated Regions and Difference in Methylation Level

We used a sliding window approach to detected DMRs, comparing the CG methylation levels between the two sample data for a window containing at least five CG (CG/CHG/CHH) sites in the same position. The area with significant difference (| fold change| ≥ 2, and Fisher’s exact test *P-*value ≤ 0.01) in methylation levels between the two samples was considered DMR. The DMRs were ranked across the genome (genome-wide scale); if the contiguous regions formed by two adjacent DMRs had significant differences of methylation levels in both samples, then the two DMRs would be merged into one continuous DMR or, otherwise, into two independent DMRs. The degree of difference in methylation level at one site in the two samples can be calculated using the following formula:

$$D={\text{log}}_{2}\,\frac{\text{Rm}\,1}{\text{Rm}\,2}$$

*D* indicates the degree of difference in methylation level, and Rm1 and Rm2 represent the methylation levels of mC of sample 1 and sample 2, respectively. If the value of Rm1 or Rm2 is 0, it is to be replaced with 0.001 (Heyn et al., 2012). We compared the 1-day sample to 0-h sample (1-day/0 h), the 3-day sample to 1-day sample (3-day/1-day), and the 5-day sample to 3-day sample (5-day/3-day) to detect DMRs for three methylation types (CG/CHG/CHH), respectively. Then, we mapped DMRs to the human reference genome (hg19) and extracted the protein-coding genes containing DMRs for further analysis.

### Enrichment of DMR to Chromatin States

Chromatin state maps data were obtained from the NIH-initiated Roadmap Epigenomics Project^2^, which we used to determine genomic location and infer DMR function. A chromatin map of 15 different states constructed from HeLa-S3 cervical cancer cells including five histone marks (H3K4me1, H3K4me3, H3K36me3, H3K27me3, and H3K9me3) was downloaded to as HeLa cell reference (Epigenome ID: E117) (Roadmap Epigenomics Project^3^). The significance and fold enrichment of DMR to each state was calculated as previously reported (Wahlberg et al., 2016).

### Genomic Annotation and Enrichment Analysis

The genome annotations were downloaded from UCSC table browser (GRCh37/hg19), the upstream, UTR5, exon, intron, UTR3, downstream, and intergenic regions were defined from annotation files. We performed Gene Ontology (GO) and KEGG pathway enrichment analyses for all DMGs with the online tool Metascape (Tripathi et al., 2015) [false discovery rate (FDR) ≤ 0.01]. The stemness enrichment analyses were carried out on the StemChecker database (Pinto et al., 2015) (*P* < 0.05). The marker genes of CSCs were collected from CSCdb (Shen et al., 2016).

### Data and Software Availability

Whole-genome DNA methylation sequencing data in this study were deposited in the Genome Sequence Archive in the BIG Data Center with the accession code (CRA001355), which are publicly accessible at http://bigd.big.ac.cn/gsa.

### Referenced Database URLs

CSCdb: http://bioinformatics.ustc.edu.cn/cscdb
Metascape: http://metascape.org/gp/index.html
StemChecker: http://stemchecker.sysbiolab.eu
SOAPnuke: see text footnote 1
Roadmap Epigenomics Project: see text footnote 2
UCSC: http://genome.ucsc.edu/
BIG Data Center: http://bigd.big.ac.cn/gsa

## Results

### Distribution of Methylated CG, CHG, and CHH in Whole Genome During TRC Selection

**FIGURE 1:**
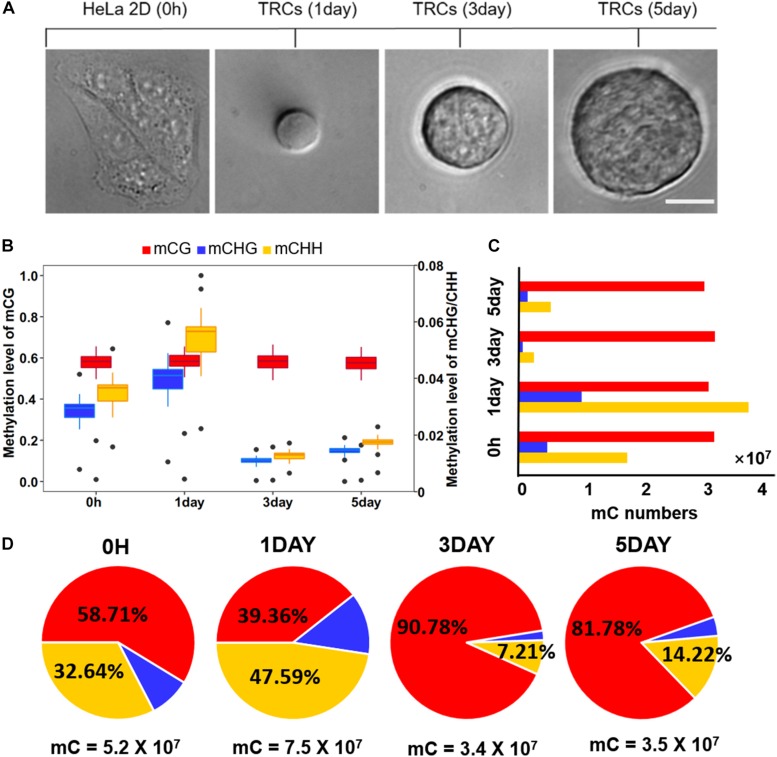
Global trends of DNA methylomes in different methylation types. **(A)** Representative images of 2D HeLa cells and multicellular tumor spheroid in 90 Pa soft fibrin gels from day 1 to day 5. Scale bar, 25 μm. **(B)** Box-plot shows the global distribution of methylation level (0–1) in each sequence context (mC in CG, CHG, CHH contexts) for all samples. The left *y*-axis indicates methylation level of mCG, while the right *y* axis represents methylation level of mCHG/mCHH. **(C)** Bar-plot shows the total numbers of mC in the CG, CHG, and CHH contexts. The methylated cytosines were counted on both strands. **(D)** The percentage of mC identified for all four samples in each sequence context. The total number of mCs for each sample is shown under the pie plot.

To investigate the dynamic changes of DNA methylation in TRC selection and transformation, WGBS (Cokus et al., 2008) was used to achieve the DNA methylomes of HeLa 2D cells (as 0 h) and HeLa 3D TRCs at three time points: 1-, 3-, and 5-day (Figure 1A). We obtained 100 GB pairs of clean data for each sample, which is 30× of the human genome, and the genome coverage of each sample average ranged 90.10–90.35%, accounting for 89.95–91.90% of cytosine in the genome (Supplementary Table S1).

Global methylation analysis revealed that CG methylation (Petrova et al., 2014) levels were similar (58%) in all time-point samples. While methylation levels of non-CG contexts (mCHG and mCHH) were significantly increased (*P* < 10^–16^, paired *t*-test) from 0 h to 1-day (Figure 1B), they suggested a dramatic methylation change on non-CG sites caused by mechanical environments (switching from 2D rigid to 3D soft). The total number of CG methylation (mCG) sites was similar (about 30 million in each sample), while the number of non-CG methylation was significantly changed over time (Figure 1C). The methylation of non-CG contexts accounted for the largest proportion (60%) in 1-day (Figure 1D), which was caused by a significant increase of non-CG mC (Figure 1C). It has been reported that non-CG methylation levels are higher in human ES cells (Thomson et al., 1998) and comprising almost 25% of all mC in H1 stem cells; but, it is absent in fibroblast cell lines (Lister et al., 2009), which implies TRCs at 1-day with strong stemness.

### Whole-Genome Methylation Levels in Different Gene Regions

**FIGURE 2:**
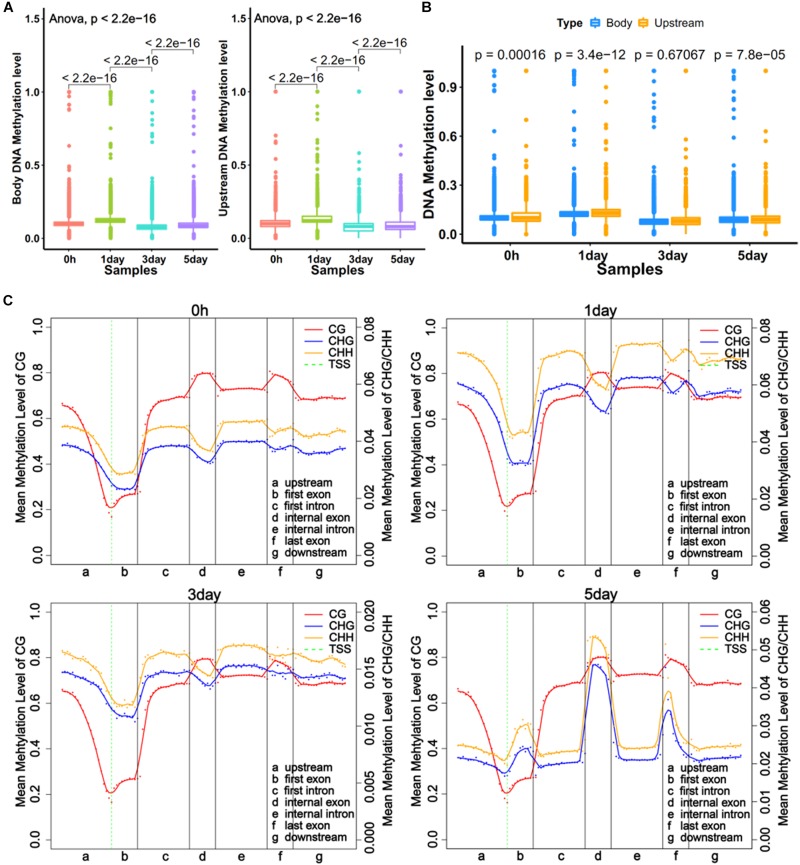
Global trends of DNA methylomes in different gene regions. **(A)** Average methylation level of different samples within upstream and gene bodies. The boxes in Graphs **A** and **B** represent quartiles, while whiskers represent minimum and maximum values. **(B)** DNA methylation level between upstream and gene body regions. **(C)** Average methylation levels throughout different gene regions in four samples. The entire gene was divided into seven different regions, denoted by the *x*-axis. The length of each transcription element region was an equal number of bins. Each dot shows the average methylation level of the bin; the full lines show the mean methylation level of five-bins. The green dotted line between Graphs **A** and **B** is TSS (transcription start site) position. Left *y*-axis indicates mean methylation level (0–1) of CG, right *y*-axis indicates methylation level of CHG or CHH.

DNA methylations in promoter and gene body regions have been reported to play different roles in gene regulation (Lou et al., 2014). We obtained the average methylation level for the upstream of 11,229 genes (2 kb upstream of transcriptional start site) and the gene body of 12,250 genes. The regions defined for genes were referred to in literature by Lou et al. (2014). The trends of methylation level in the upstream and the body were very similar at four time points, with the 1-day sample showing the highest level (Figure 2A). The results also showed significant differences (*P* < 0.001) in methylation levels between adjacent time point samples (Figure 2A). The comparison showed that the methylation level of the gene body was a little bit higher (*P* < 0.001) than upstream region in three samples (Figure 2B). However, the distribution of DNA methylation levels in different gene regions (Li et al., 2010) revealed that mCG levels of different gene regions did not vary with time, but the non-CG methylation varied greatly among different samples (Figure 2C). This was consistent with a report from Shirane et al. (2013). The result showed the degrees of non-CG methylation was highest in 1-day samples, lowest in 3-day samples, and slightly elevated in 5-day samples, which is the same trend in all exons (first, internal, and last exon) (Figure 2C). Interestingly, the CHH methylation levels were always higher than CHG (Figure 2C). It was demonstrated that non-CG DNA methylation plays an important role in the selection of TRCs.

### Differentially Methylated Regions in TRC Selection

**FIGURE 3:**
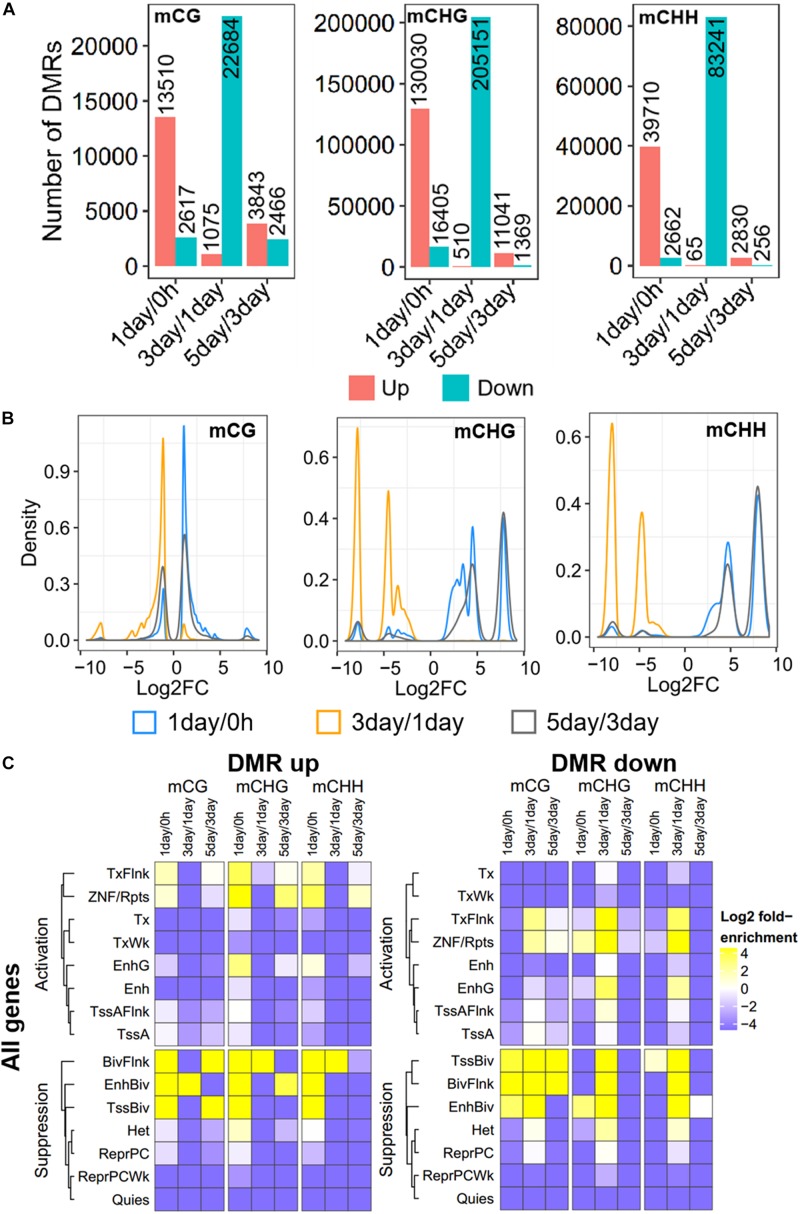
Distribution and annotation of differentially methylated regions. **(A)** Number distribution of DMRs. DMRs were categorized into three groups: 1-day versus 0 h (1-day/0 h), 3-day versus 1-day (3-day/1-day), and 5-day versus 3-day (5-day/3-day). Up indicates increased methylation in comparison, while down represents decreased. **(B)** Density plots of degree of differential methylation among DMRs. Log_2_FC of methylation levels between two samples for each DMR were shown as *x*-axes. DMRs: differentially methylated regions. **(C)** Enrichment of DMRs for all genes to chromatin states in HeLa-S3 cells. The fold enrichment was calculated from the observed base overlap and state divided by the genome-wide expected fraction for each state. Color key from purple to yellow indicates the Log_2_ fold-enrichment from low to high. DMR up indicates increased methylation in comparison, while down represents decreased. The chromosome with 15 different chromatin states, including 8 activation states and 7 suppression states. TssA, active TSS; TssAFlnk, flanking active TSS; TxFlnk, transcr. at gene 5′ and 3′; Tx, strong transcription; TxWk, weak transcription; EnhG, genic enhancers; Enh, enhancers; ZNF/Rpts, ZNF genes and repeats; Het, heterochromatin; TssBiv, bivalent/poised TSS; BivFlnk, flanking bivalent TSS/Enh; EnhBiv, bivalent enhancer; ReprPC, repressed PolyComb; ReprPCWk, weak repressed PolyComb; Quies, quiescent/low.

Further, to compare DNA methylation in TRC selection, we identified the DMRs of mCHG, mCHH, and mCG, and compared them in three stages: 1-day versus 0 h (1-day/0 h), 3-day versus 1-day (3-day/1-day), and 5-day versus 3-day (5-day/3-day) (Figure 3A), using stringent criteria (section “Materials and Methods”). We identified 539,465 DMRs in all comparisons, and about 92% of DMRs were in non-CG contexts. The number of CHG DMRs (364,506) and CHH DMRs (128,764) were much larger than that of the CG DMRs (46,195). Interestingly, >85% of DMRs showed an increase in methylation from 0 h to 1-day or from 3- to 5-day in all CG contexts. On the contrary, the majority of DMRs (95–99%) were decreased in methylation from 1- to 3-day (Figure 3A). Furthermore, the number of DMRs of 3-day/1-day accounts for half of total DMRs. The DMR distribution of chromosomes is shown in Supplementary Figure S1.

The methylation level difference of each DMR between two samples was measured by calculated fold change (FC). Although the ratios of hypermethylation and hypomethylation DMRs in the three methylation types were similar, the FC of differential methylation varied widely across different methylation types (Figure 3B). The density plots of log_2_FC showed that the difference in non-CG methylation exhibited a bimodal pattern at all three stages, and log_2_FC of most of mCHG/mCHH DMR was >5, while CG methylation had a single peak, except in 5-day/3-day, and the log_2_FC of >90% of mCG DMRs was <3 (Figure 3B). The difference of DMRs in non-CG contexts was greater than that in mCG, implying that non-CG DMR played a critical role in TRC selection.

To make a detailed annotation of DMRs, we used chromatin state maps from HeLa-S3 cells (Figure 3C; Kundaje et al., 2015) and carried out enrichment analyses of DMRs of each stage. The DMRs with elevated methylation levels were not enriched in chromatin activation states such as strong transcription (Tx), weak transcription (TxWk), flanking active TSS (TssAFlnk), and active TSSTssA (TssA), but were significantly enriched in chromatin inhibition states including flanking bivalent TSS/Enh (BivFlnk), bivalent enhancer (EnhBiv), and bivalent/poised TSS (TssBiv) in all three methylation types at 1-day/0-h stage. For DMRs with a decreased methylation level, non-CG methylated DMRs were enriched in more regions predicted to be chromatin-activated than in mCG in 3-day/1-day samples, such as region EnhG/Enh and transcription-related region Tx (Figure 3C). These suggests the potential role of non-CG methylation of these functional regions.

### Methylation Pattern and Functional Analysis of DMGs

**FIGURE 4:**
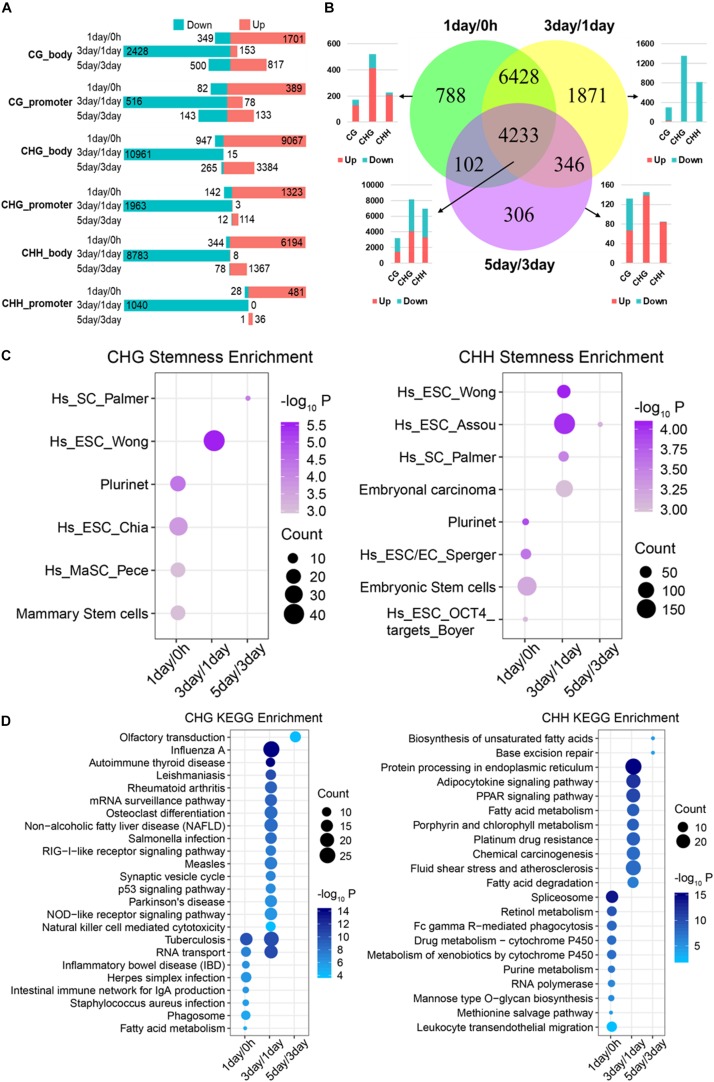
Functional analysis acid of stage-specific differentially methylated genes. **(A)** Distribution of DMGs in different groups. Red and cyan in parts **A** and **B** indicate that the methylation level is increased or decreased in comparison. **(B)** Venn-like diagram shows overlapping DMGs among three stages. The bar plot shows the number of each stage-specific DMGs and the three-stage common DMGs (Bottom left). **(C)** Stemness enrichment analysis (*P* < 0.05) of stage-specific DMGs of CHG (Left) and CHH (Right) methylation. **(D)** Significantly enriched terms (FDR < 0.01) in KEGG pathway for stage-specific DMGs of CHG (Left) and CHH (Right) methylation. CC, cellular component; BP, biological process; MF, molecular function.

To study DMGs for different stages, we mapped DMRs to protein-coding genes (Figure 4A). DMGs of mCG in 3-day/1-day stage accounted for approximately 70% of total DMGs for all stages, while the ratios were 90% for mCHG and 88% for mCHH, suggesting a dramatic change in methylation at 3-day/1-day in TRC screening. Besides, the numbers of DMGs and DMRs of mCG were less than those of non-CG at any stage (Figures 3A, 4A), which indicated that only a small number of mCG sites were affected during reprogramming or dedifferentiation, consistent with previously reported (Nishino et al., 2011).

We identified that 788, 1,871, and 306 genes are stage-specific DMGs for 1-day/0 h, 3-day/1-day, and 5-day/3-day, separately (Figure 4B). Approximately 82% of the 1-day/0 h-specific DMGs had significantly higher methylation levels when compared to 1-day with 0 h. In contrast, 98% of 3-day/1-day specific DMGs changed from a “hypermethylated” state in 1-day into a “hypomethylated” state in 3-day. Then, methylation level of 81% of the DMGs increased dramatically from 3- to 5-day. Consistent with stage-specific DMGs, the methylation level of most (>90%) of common DMGs shared in the three stages (Figure 3B) increased from 0 h to 1-day (Supplementary Figure S2A), reduced from 1- to 3-day (Supplementary Figure S2B), and then raised from 3- to 5-day (Supplementary Figure S2C). These were consistent with the DMRs that shown in Figure 3A. Interestingly, the majority of the hypo-methylated of 1-day/0 h and 3-day/1-day were non-CG methylation, whereas the majority of the hypo-methylated of 3-day/5-day were mCG (Figure 4B and Supplementary Figures S2A–C).

Since most of the stage-specific DMGs are derived from non-CG methylation (Figure 4B and Supplementary Figure S2D), we performed stemness, KEGG pathway enrichment analyses (Figures 4C,D) to further investigate functions of stage-specific DMGs of non-CG during the selection of TRCs. Stemness enrichment analysis indicates that stage-specific DMGs of non-CG methylation were significantly enriched in the stem cell or CSC marker gene sets (Figure 4C). KEGG pathway results demonstrated that CHG DMGs in 3-day/1-day were associated with “Osteoclast differentiation” and “p53 signaling pathway,” while CHH DMGs were enriched in “Chemical carcinogenesis” and “PPAR signaling pathway” in 3-day/1-day (Figure 4D). Furthermore, stemness, KEGG pathway enrichment analyses of all three stage-specific DMGs showed that these DMGs were associated with stem cell or CSC marker gene sets (Supplementary Figure S2E) and 3-day/1-day stage-specific DMGs were enriched in many immune and differentiation-related pathways such as “Autoimmune thyroid disease,” “Th1 and Th2 cell differentiation,” and “Osteoclast differentiation” (Supplementary Figure S2F). These indicated that many DMGs of non-CG in 3-day/1-day phase were involved in cell differentiation during TRC selection. GO analysis indicated that genes in these three stages were involved in oxidoreductase activity, autophagy, inflammatory response, and cell differentiation (Supplementary Figure S2G). For 4,233 common DMGs (Figure 4B), the results showed that 4,225 common DMGs were derived from non-CG methylation; Venn plot indicated that 2,173 DMGs only contain non-CG DMRs, but eight genes consist of only mCG DMRs (Supplementary Figure S3A). Further, pathway enrichment analysis (Supplementary Figure S3B) revealed that the non-CG-specific DMGs of three-stage common genes were involved in many cancer-related pathways and hundreds of DMGs were related to cancer stemness (Supplementary Figure S3C). These results suggested that stage-specific/common DMGs of non-CG methylation affecting cell stemness and cell differentiation may play important roles in the TRC screening process.

### Methylation Pattern Analysis of CSC Marker Genes in DMGs

**FIGURE 5:**
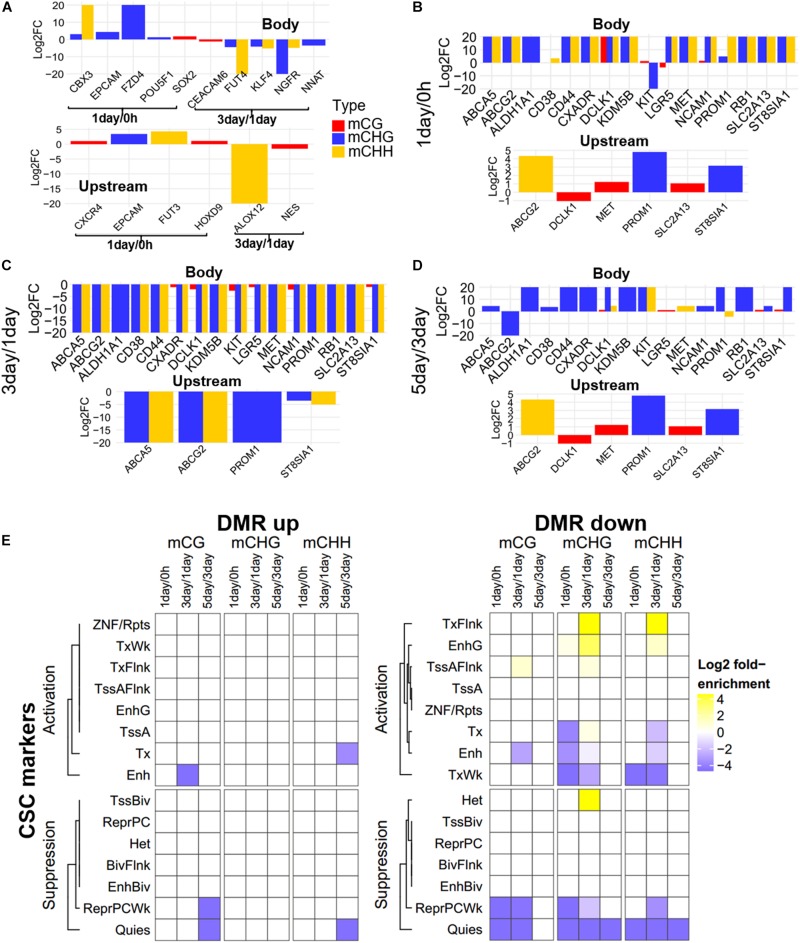
Methylation pattern of CSC marker genes among DMGs. **(A)** Methylation differences of CSC marker genes in stage-specific DMGs in gene body and upstream region (Bottom). FC, fold change. **(B–D)** Methylation differences of CSC marker genes as three-stage common DMGs at 1-day/0-h stage **(B)**, 3-day/1-day stage **(C)**, and 5-day/3-day stage **(D)**. **(E)** Enrichment of DMRs for CSC marker genes to chromatin states in HeLa-S3 cells. The fold enrichment was calculated from the observed base overlap and state divided by the genome-wide expected fraction for each state. Color key from purple to yellow indicates the Log_2_ fold-enrichment from low to high. DMR up indicates increased methylation in comparison, while down represents decreased.

Focusing on the CSC marker genes, we found that eight CSC marker genes (*CBX3*, *CXCR4*, *EPCAM*, *FUT3*, *FZD4*, *HOXD9*, *POU5F1*, and *SOX2*) were in 1-day/0 h-specific DMGs, seven (*ALOX12*, *CEACAM6*, *FUT4*, *KLF4*, *NES*, *NGFR*, and *NNAT*) were unique in 3-day/1-day (Figure 5A), and 16 marker genes belonged to three-stage common DMGs (Figures 5B–D). These CSC marker genes had significant changes in non-CG methylation in all three stages, but had little changes in mCG, whether in the gene body or promoter region (Figure 5). Furthermore, only four to six DMGs of CSC marker genes had methylation changes in upstream regulatory region, confirming methylation changes of these genes were more likely to occur in the gene body region. The expression levels of mechanotransduction genes that were changed significantly from HeLa 2D to HeLa TRCs in 3-day samples in the previous project (Huang et al., 2019) here were also changed significantly in non-CG methylation (Supplementary Figure S4). The enrichment of DMRs contained in stemness marker genes in each chromatin state region was further analyzed (Figure 5E). It was found that the DMRs with increased methylation levels were not significantly enriched at all stages. The DMRs with decreased methylation levels were significantly enriched in some regions including TssAFlnk, actively transcribed region (TxFlnk), and gene bodies enhancers (EnhG) only at 3-day/1-day, and the enrichment of chromatin activation status regions of non-CG methylation was more than mCG.

**FIGURE 6:**
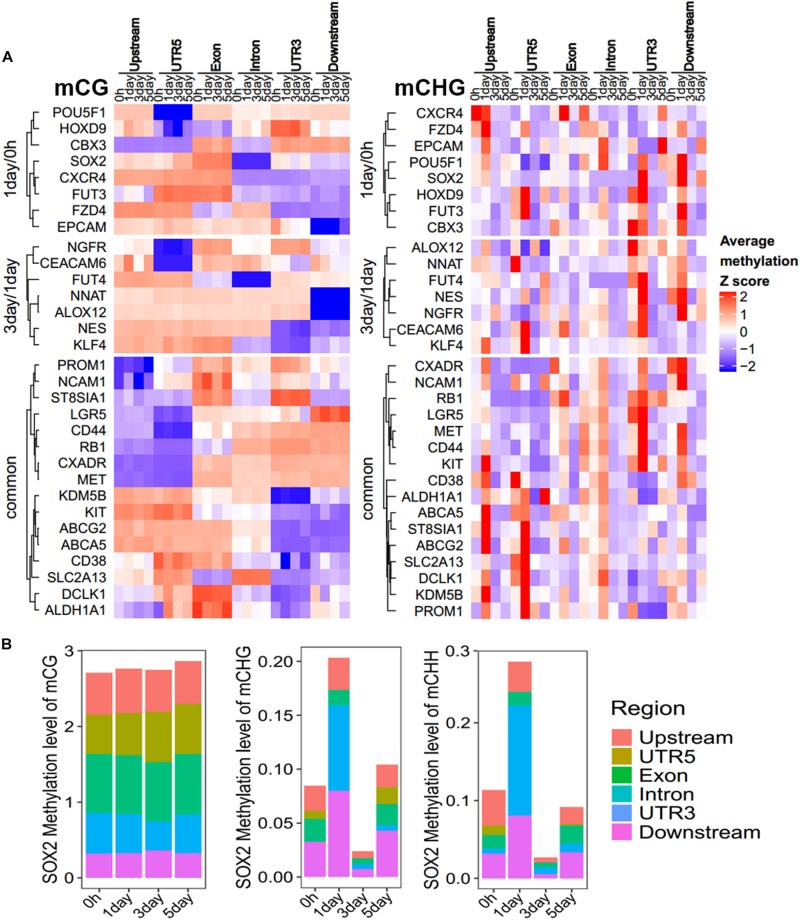
Methylation pattern of CSC marker genes. **(A)** Heatmap showing the DNA methylation trend of CSC marker genes for mCG/mCHG, the methylation level of each gene in each row was normalized by *Z*-score. These genes were categorized into three groups: 1-day/0 h (unique in 1-day/0-h stage), 3-day/1-day (unique in 3-day/1-day stage), and common (common DMGs in three stages). Each class of genes was performed in hierarchical clustering according to Euclidean distance. Scale from blue to red indicates the normalized methylation level from low to high. **(B)** Stacked bar-plots show the SOX2 methylation level in gene regions. The *y*-axis shows the sum of methylation levels in all regions.

To further explore which gene regions determine the methylation pattern of these genes, we analyzed the methylation profiles within marker genes in Figure 5, among different regions (Figure 6A, Supplementary Figure S5, and Supplementary Table S4). All these genes had less change in CG methylation levels at various time points in a certain region (Figure 6A), but the non-CG methylation levels changed greatly (Figure 6A and Supplementary Figure S5), especially in the UTR5, UTR3, and downstream regions. For example, the CSC marker gene SOX2 acts as one of the unique DMGs in 1-day/0 h and showed significant differences of CHG/CHH methylation in UTR5, UTR3, and downstream regions, but no significant difference was observed in CG methylation (Figure 6B). The same as with chromatin state regions, little changes were observed in mCG of marker genes, but non-CG methylation level of TssAFlnk, TxWk, and Enhancers (Enh) fluctuated greatly (Supplementary Figure S6). These results showed that most of these CSC marker genes were biased toward changing its methylation in non-CG methylation and gene body regions.

### DNA Methylation of Continuously Changed Genes During Three Stages

**FIGURE 7:**
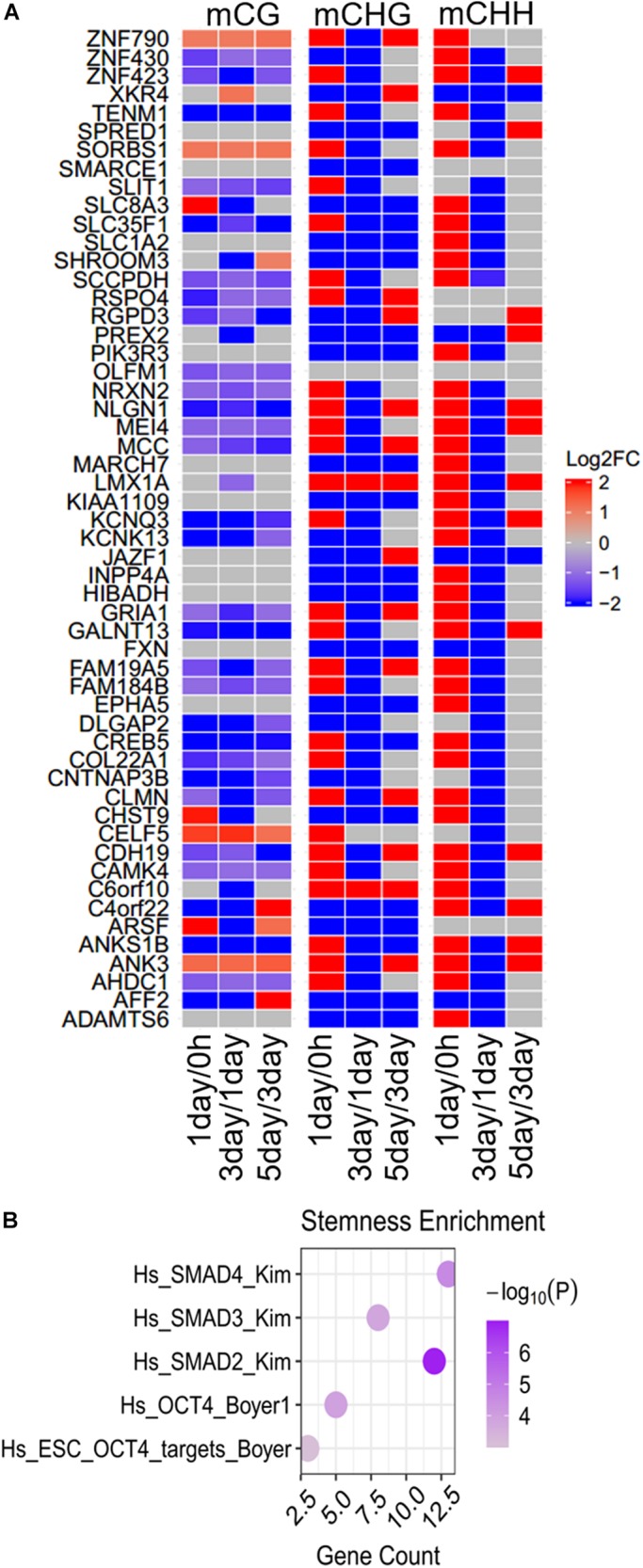
Differences and enrichment analysis of continuously changed genes in DNA methylation. **(A)** Heatmap shows the methylation differences of continuously changed genes in the three stages. Scale from blue to red indicates the log_2_FC from low to high. **(B)** Stemness enrichment analysis of continuously changed genes. The significant terms (FDR < 0.01) are shown in the figure.

The modifications of continuously hyper- or hypomethylated CG dinucleotides provide a biomarker for replicative senescence (Franzen et al., 2018). We identified 54 genes whose methylation levels were continuously increased or decreased from 0 h to 5 days in at least one methylation type (Figure 7A). Thirty-eight genes belonging to them were continuously changed (5 genes continuously increased, and 33 genes continuously decreased) in mCG, 20 genes continuously changed in mCHG, and only 2 genes (*JAZF1* and *XKR4*) continuously changed in mCHH. The methylation altered in these genes may affect tumor proliferation; for instance, the *CREB5* continuously decreased in mCG (Figure 7A) was hypomethylated in Graves’ disease (Cai et al., 2015). Hypermethylation of *ADAMTS6* is identified in multiple cancers (Kordowski et al., 2018), and its methylation level continuously decreased in mCHG (Figure 7A), while *LMX1A* continuously increased in mCHG and serve as a DNA methylation marker in cervical cancer (Lai et al., 2008).

Same as the specific DMGs and common DMGs as mentioned above, most (85.2%) of the 54 genes at first were increased and then decreased, but at last increased at three stages in at least one non-CG methylation (mCHG/mCHH). This is a hallmark of the methylation in the TRC screening process. Although more mCG DMGs could be observed in most (ca. 75%) of continuously changed genes, differences in non-CG methylation were more significant (| log_2_FC| > 20) than in mCG (| log_2_FC| < 5). None of the continuously changed genes were CSCs marker genes, but were notably enriched in five stemness-related gene sets (Figure 7B), indicating their importance in cancer cell stemness.

### Functional Analysis of Three Types of DMGs at Different Stages

**FIGURE 8:**
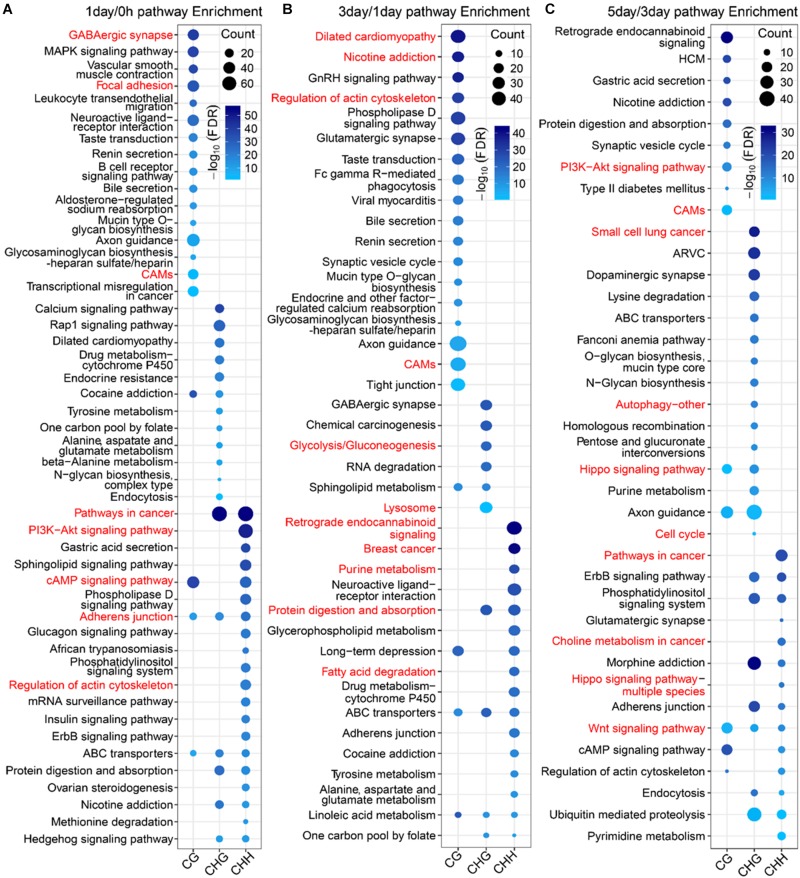
Pathway enrichment analysis of three types of DMGs at different stages. **(A–C)** Significantly enriched terms (FDR < 0.01) in KEGG pathway for three types of DMGs at stages of 1-day/0 h **(A)**, 3-day/1-day **(B)**, and 5-day/1-day **(C)**. CAMs, cell adhesion molecules; HCM, hypertrophic cardiomyopathy; ARVC, arrhythmogenic right ventricular cardiomyopathy.

To further explore the difference in the function between mCG DMGs and non-CG DMGs in the TRC-selecting process, we compared here the functions of different methylation types of DMGs at the same stage. The number of DMGs for three types of DMGs at three stages is shown in Supplementary Table S3. For 1-day/0 h, DMGs of mCG were significantly (FDR < 0.01) enriched in the brain-related pathway (Figure 8A), such as the “GABAergic synapse,” as well as mechanical transduction-related pathways, such as “focal adhesion” and “cell adhesion molecules (CAMs).” DMGs of mCHG and mCHH were significantly enriched in mechanical transduction-related pathways such as “PI3K-Akt signaling pathway,” “regulation of actin cytoskeleton,” and “adherens junction.” In the 3-day/1-day phase, the most significant pathway for mCG and mCHG/mCHH was neurologically related (Figure 8B), which is consistent with the report that non-CG sites were most frequently methylated in the human brain (Varley et al., 2013). Besides, genes of mCG were enriched in the “regulation of actin cytoskeleton pathway.” During the 5-day/3-day phase (Figure 8C), it was found that both mCG and non-CG DMGs were enriched in the WNT pathway, and DMGs of non-CG methylation were enriched in pathways associated with cancer and mechanical transduction including WNT, Hippo, cell cycle, autophagy, pathways in cancer, and so on. These results indicated that many DMGs of non-CG methylation are involved in proliferation and differentiation of cancer and play vital roles during the TRC selection.

## Discussion

Previous studies have reported that DNA methylation has effects on cancer cell differentiation or reprogramming (Lister et al., 2009, 2011; Kulis et al., 2015). However, DNA methylation variation and impacts in the TRC screening process have rarely been studied. In this study, we performed WGBS and revealed the highly dynamic nature of DNA methylation during 3D culturing process. Profiling of different regulatory regions and patterns of CG and non-CG methylation of CSC marker genes suggest possible different roles for DNA methylation in cancer stemness. To the best of our knowledge, this is the first study of DNA methylation dynamics changes in TRC selection.

Our results showed that DNA methylation level of whole-genome (Figure 1B) and regulatory regions (Figure 2C) of mCG were similar in each sample but showed significantly changed of non-CG methylation at different time points (Figures 1B, 2C). These imply little dynamic changes in CG methylation during TRC selection, which is consistent with previously reported that only a few CG sites are affected during reprogramming (Nishino et al., 2011). Some studies have previously detected non-CG methylation in human cells, particularly in embryonic stem cells (Ramsahoye et al., 2000; Lister et al., 2009; Ziller et al., 2011). Non-CG methylation disappeared in differentiated cells and had a high level in induced pluripotent stem cells (Lai et al., 2008) and human embryonic stem cells (Laurent et al., 2010). We found that the number of mC sites and the methylation level of non-CG context were significantly increased in 1-day samples but decreased in 3-day samples (Figures 1C, 3B). These suggested TRCs in 1-day samples might be the most CSC-like cells. Furthermore, methylation levels of CSCs markers showed significant increase and then decrease, at last slightly restored during the process of 3D culture (Figures 5A–D), changing greatly in non-CG methylation, especially in the UTR5, UTR3, and downstream regions (Figure 6 and Supplementary Figure S5). It may be that the non-CG methylation is needed to reestablished after cell division to maintain, this being consistent with previous hypothesis (Ichiyanagi et al., 2013; Patil et al., 2014). Chromatin status by chromosomal region was inferred by cross-referencing to states of HeLa-S3 cervical cancer cells from the Roadmap Epigenomics Project; we found that non-CG DMRs were more likely to be enriched in the activated chromatin status (Figures 3C, 5C), and the non-CG methylation changed greatly in actively transcribed regions such as TssAFlnk and Enh regions (Supplementary Figure S6). We speculated this methylation pattern of non-CG methylation may be a feature of DNA methylation during TRC screening.

Previous studies have reported that mechanical stretch or soft matrix significantly decreased the DNA methylation level of critical CG sites or specific genes (Vlaikou et al., 2017; Wang et al., 2017; Pennarossa et al., 2018), and non-CG methylation may regulate gene expression according to environmental changes (Patil et al., 2014). Our results were consistent with these reports that numerous DMRs and DMGs of mCG and non-CG methylation were identified in the stage (1-day/0 h) that environment-switched from 2D rigid to 3D soft (Figure 4A and Supplementary Figure S1). Furthermore, 82% of DMGs had significantly higher methylation levels (Figure 3A) in 1-day samples when compared with 0 h, which was supported by the report that higher DNA methylation levels were detected in pancreatic CSCs (Zagorac et al., 2016). Besides, the number of non-CG DMGs was greater than CG DMGs (Figure 3A), and mechanotransduction genes were changed significantly in non-CG methylation (Supplementary Figure S4); we speculate that non-CG methylation is more affected by mechanical forces, although few studies have been conducted so far. The functional analysis showed that many DMGs of the non-CG methylation are involved in cancer proliferation and cell differentiation, while DMGs of mCG are associated with brain nerve, synaptic membrane, cytoskeleton, and membrane transport (Figure 8). This suggested that non-CG methylation has a greater effect than CG in cancer stemness, which may affect the TRC screening process.

## Conclusion

In conclusion, we demonstrated that DNA methylation changes in TRC selection explored the specific methylation pattern for CSC marker genes, and found a significant difference between DMGs of CG and non-CG. Essential future studies will need to investigate how mechanical forces affect non-CG methylation, whether the change of non-CG methylation is a cause or consequence of cell stemness, and whether its dynamics can indicate different states of cells in selection of TRCs.

## Data Availability Statement

The datasets generated for this study can be found in the whole genome DNA methylation sequencing data which were deposited to the BIG Data Center with the accession code (CRA001355), which is publicly accessible at http://bigd.big.ac.cn/gsa.

## Author Contributions

NW, XY, and A-YG were responsible for study conception and design. WH and HH were responsible for acquisition of data. WH, HH, and QZ were responsible for data analysis. WH, HH, and A-YG were responsible for drafting and revision of the manuscript.

## Conflict of Interest

The authors declare that the research was conducted in the absence of any commercial or financial relationships that could be construed as a potential conflict of interest.

## Footnotes

1 https://github.com/BGI-flexlab/SOAPnuke

2 www.roadmapepigenomics.org

3 http://egg2.wustl.edu/
