# Supplementary material for: Genome-Wide DNA Methylation Enhances Stemness in the Mechanical Selection of Tumor-Repopulating Cells
Source: Front Bioeng Biotechnol. 2020 Mar 17;8:88. doi: 10.3389/fbioe.2020.00088 (PMC7090028; doi:10.3389/fbioe.2020.00088)
Supplement: Supplementary file 1 [file Presentation_1.pdf]

frontiers

Supplementary Material

Genome-wide DNA methylation enhance stemness in mechanical selecting tumor-repopulating cells

Wei Huang[#1], Hui Hu[#2], Qiong Zhang[2], Ning Wang[3,4], Xiangliang Yang[1*], An-Yuan Guo[2*]

[*] Correspondence: guoay@hust.edu.cn (A.Y.G.), yangxl@hust.edu.cn (X.Y.)

This file includes:

Tables S1, S2, S3 and S4

Figures S1, S2, S3, S4, S5 and S6

1.1 Supplementary Figures

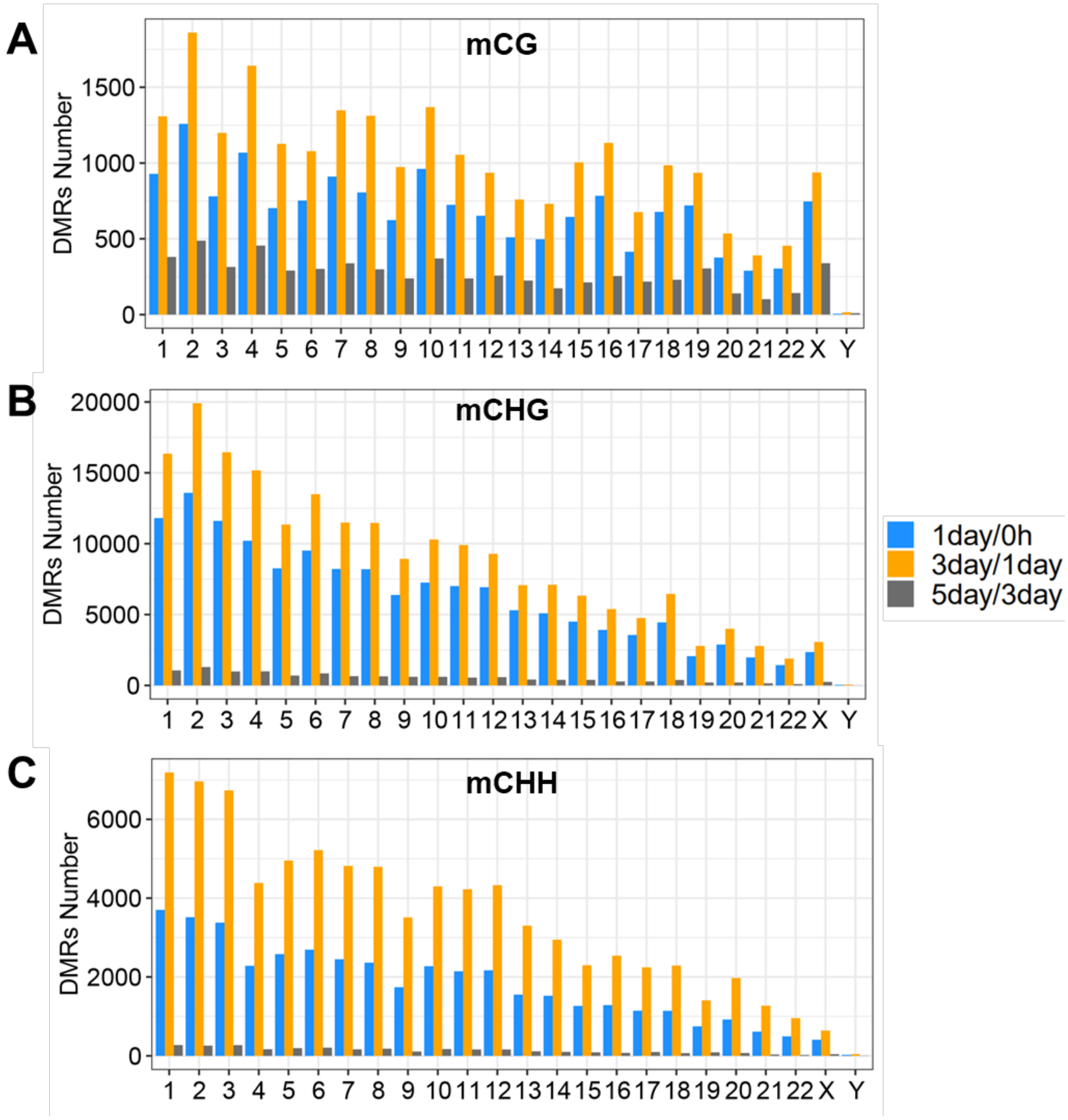

Supplementary Figure S1. DMR distribution among different sequence context of three stage.

The bar plot shows the number of DMRs of three sequence contexts (CG/CHG/CHH) on individual chromosome at different stages.

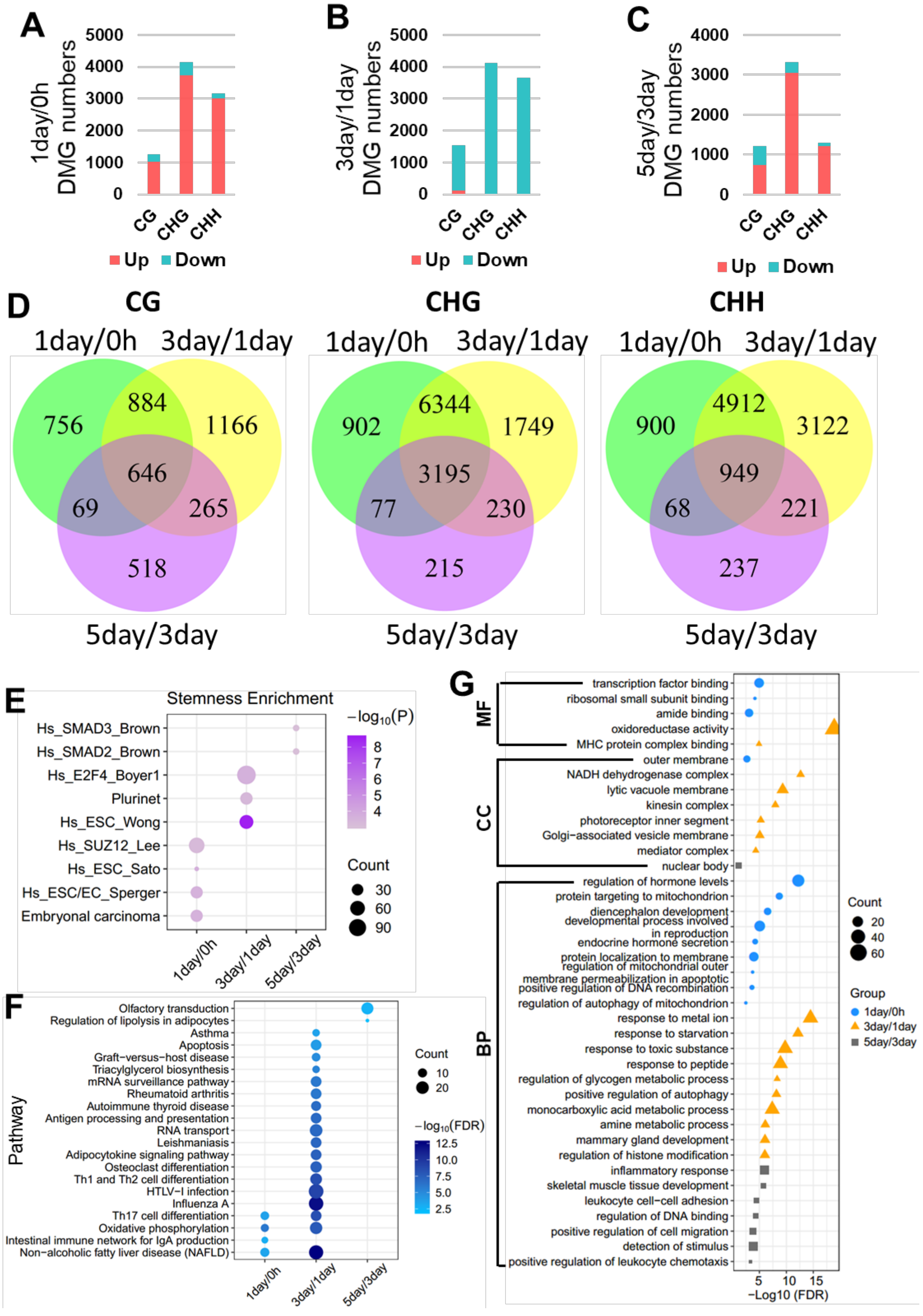
A
1day/0h DMG numbers
5000
4000
3000
2000
1000
0
CG CHG CHH
Up Down
B
3day/1day DMG numbers
5000
4000
3000
2000
1000
0
CG CHG CHH
Up Down
C
5day/3day DMG numbers
4000
3000
2000
1000
0
CG CHG CHH
Up Down
D
CG
1day/0h 3day/1day
756 884 1166 646 69 265 518
5day/3day
CHG
1day/0h 3day/1day
902 6344 1749 3195 77 230 215
5day/3day
CHH
1day/0h 3day/1day
900 4912 3122 949 68 221 237
5day/3day
E
Stemness Enrichment
Hs_SMAD3_Brown
Hs_SMAD2_Brown
Hs_E2F4_Boyer1
Plurinet
Hs_ESC_Wong
Hs_SUZ12_Lee
Hs_ESC_Sato
Hs_ESC/EC_Sperger
Embryonal carcinoma
1day/0h 3day/1day 5day/3day
−log10(P)
8 7 6 5 4
Count
30 60 90
F
Pathway
Olfactory transduction
Regulation of lipolysis in adipocytes
Asthma
Apoptosis
Graft−versus−host disease
Triacylglycerol biosynthesis
mRNA surveillance pathway
Rheumatoid arthritis
Autoimmune thyroid disease
Antigen processing and presentation
RNA transport
Leishmaniasis
Adipocytokine signaling pathway
Osteoclast differentiation
Th1 and Th2 cell differentiation
HTLV−I infection
Influenza A
Th17 cell differentiation
Oxidative phosphorylation
Intestinal immune network for IgA production
Non−alcoholic fatty liver disease (NAFLD)
1day/0h 3day/1day 5day/3day
Count
10 20
−log10(FDR)
12.5 10.0 7.5 5.0 2.5
G
MF
transcription factor binding
ribosomal small subunit binding
amide binding
oxidoreductase activity
MHC protein complex binding
CC
outer membrane
NADH dehydrogenase complex
lytic vacuole membrane
kinesin complex
photoreceptor inner segment
Golgi−associated vesicle membrane
mediator complex
nuclear body
BP
regulation of hormone levels
protein targeting to mitochondrion
diencephalon development
developmental process involved in reproduction
endocrine hormone secretion
protein localization to membrane
regulation of mitochondrial outer membrane permeabilization in apoptotic
positive regulation of DNA recombination
regulation of autophagy of mitochondrion
response to metal ion
response to starvation
response to toxic substance
response to peptide
regulation of glycogen metabolic process
positive regulation of autophagy
monocarboxylic acid metabolic process
amine metabolic process
mammary gland development
regulation of histone modification
inflammatory response
skeletal muscle tissue development
leukocyte cell−cell adhesion
regulation of DNA binding
positive regulation of cell migration
detection of stimulus
positive regulation of leukocyte chemotaxis
5 10 15
−Log10 (FDR)
Count
20 40 60
Group
1day/0h
3day/1day
5day/3day

Supplementary Figure S2. Distribution and function of stage-common/specific DMGs at different stages.

(**A-C**) The bar plots in **A, B** and **C** show the number of the three-stage common DMGs for 1day/0h (**a**), 3day/1day (**B**) and 5day/3day (**C**). Up indicates increased methylation in comparison, while down represents decreased. (**D**) Venn-like diagram shows overlapping DMGs among three stages for each methylation type. (**E**) Stemness enrichment analysis ($P < 0.05$) of stage-specific DMGs in all methylation type. (**F**, **G**) Significantly enriched terms ($FDR < 0.01$) in KEGG pathway (**F**) and Gene Ontology (**G**) for stage-specific DMGs. FDR: false discovery rate. CC: cellular component. BP: biological process. MF: molecular function.

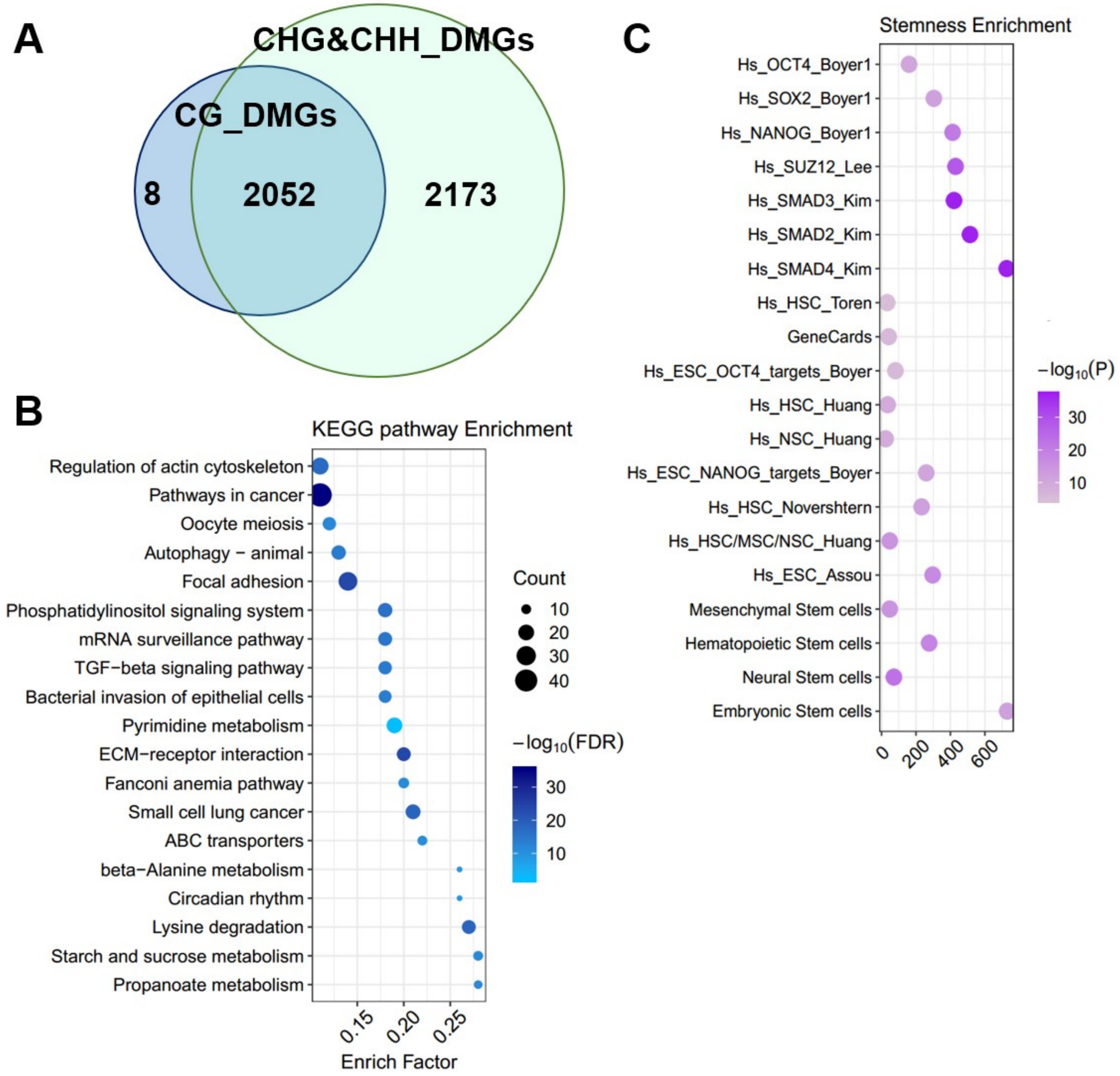

Supplementary Figure S3. Enrichment analysis of DMGs unique in non-CG methylation.

(**A**) Venn diagram shows the overlapping DMGs between CG context and non-CG contexts (CHG and CHH). (**B**) Significantly enriched terms (FDR < 0.01) in KEGG pathway for DMGs unique in non-CG contexts in **A.** (**C**) Stemness enrichment analysis of common DMGs. Only the significant terms (P < 0.01) are shown in the figure.

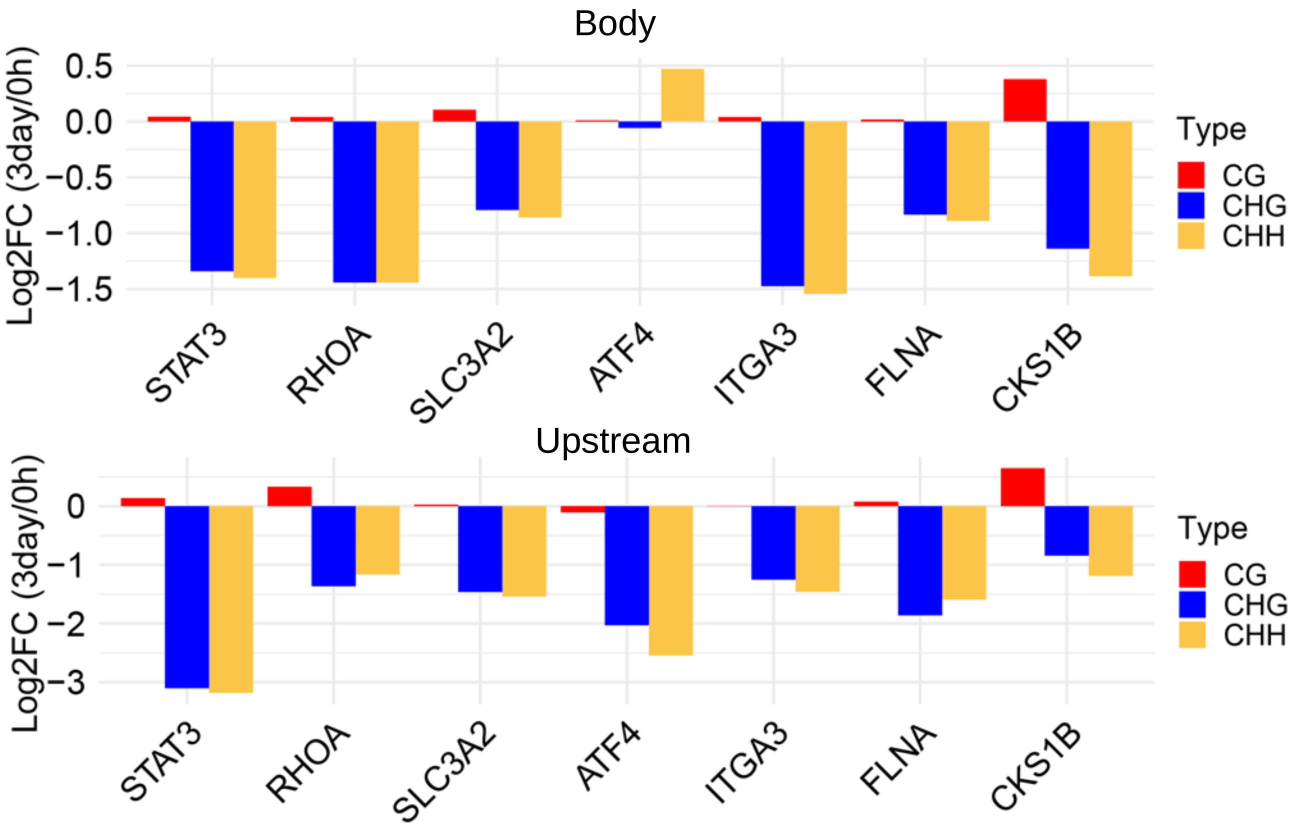

Supplementary Figure S4. Methylation pattern of mechanotransduction genes.

Methylation differences of mechanotransduction genes in comparison group 3day/0h in gene body (up) and upstream region (bottom). FC: Fold Change.

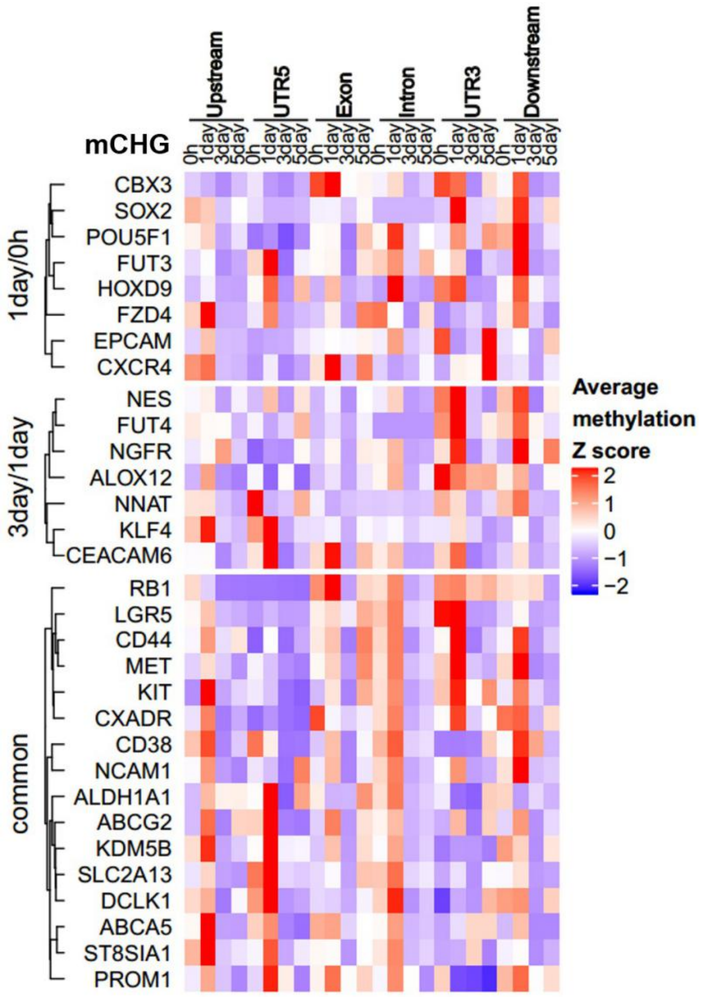

Supplementary Figure S5. DNA methylation trend of CSC marker genes for mCHH.

Heatmap showing DNA methylation trend of CSC marker genes for mCHH, the methylation level of each gene in each row was normalized by Z score. These genes are categorized into three groups: 1day/0h (unique in 1day/0h stage), 3day/1day (unique in 3day/1day stage) and common (common DMGs in three stages). Each class of genes was performed hierarchical clustering according to Euclidean distance. Scale from blue to red indicates the normalized methylation level from low to high.

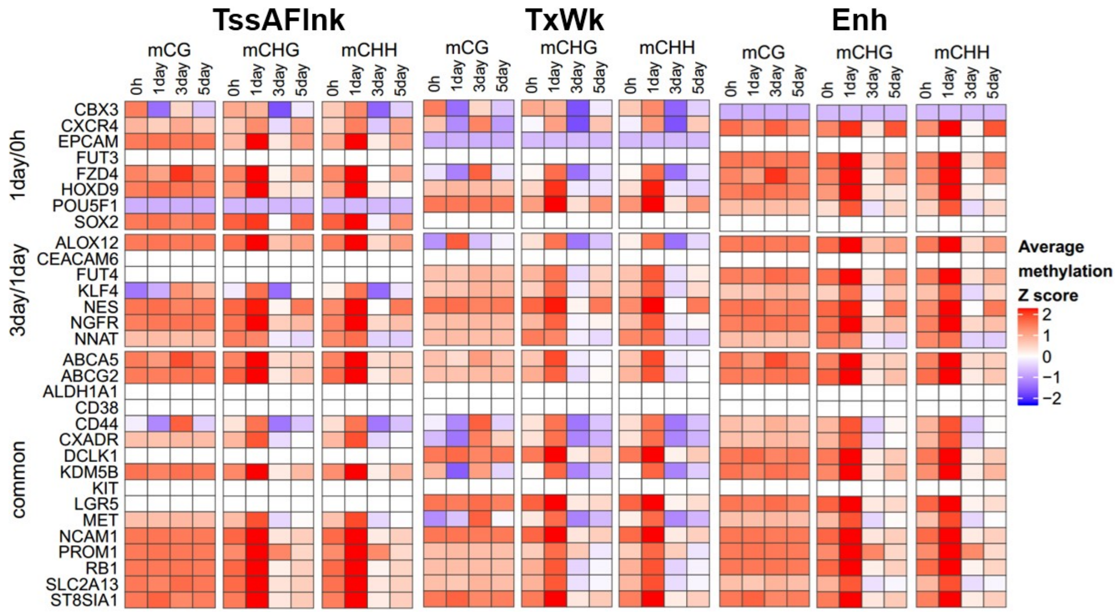

Supplementary Figure S6. DNA methylation level of CSC marker genes in different chromatin states.

Heatmap showing DNA methylation level of CSC marker genes for mCG/mCHG/mCHH in three chromatin states, the methylation level of each gene in each row was normalized by Z score. These genes are categorized into three groups: 1day/0h (unique in 1day/0h stage), 3day/1day (unique in 3day/1day stage) and common (common DMGs in three stages). Each class of genes was performed hierarchical clustering according to Euclidean distance. Scale from blue to red indicates the normalized methylation level from low to high. TssAFlnk: Flanking Active TSS; TxWk: Weak transcription; Enh: Enhancers.

1.2 Supplementary Tables

Supplementary Table S1. Reads statistics and quality control of whole genome bisulfite sequencing.

Sample ID	Clean Reads	Clean Data Size (bp)	Clean Reads Q20 Rate (%)	Bisulfite Conversion Rate (%)	Dupli-cation Rate (%)	Average Depth (X)	Coverage (%)	C Coverage (%)	Run Name	Run Acces-sion
0h	666,666,674	100 G	97.70	99.62	15.16	22.03	90.35	91.90	S0h_WGBS-Seq	CRR044932
1day	666,666,674	100 G	97.40	99.61	15.15	21.90	90.33	90.83	S1day_WGBS-Seq	CRR044933
3day	666,666,668	100 G	98.22	99.55	13.79	21.89	90.20	91.62	S3day_WGBS-Seq	CRR044934
5day	666,666,672	100 G	97.99	99.60	17.24	21.44	90.10	89.95	S5day_WGBS-Seq	CRR044935

Note: Clean Reads Q20: reads low-quality bases (quality value ≤ 20 and the ratio of low-quality base more than 10%) were removed from the raw data. Clean Rate (%) = Clean Data Size (bp)/Raw Data Size (bp); Bisulfite Conversion Rate = 100% − methylation rate of Lambda DNA. The raw read files of each sample were available by the BIG Data Center under the accession code CRA001355 (http://bigd.big.ac.cn/gsa). The run name and accession id for each sample were shown in the table.

Supplementary Table S2. Mapping result of whole genome bisulfite sequencing.

Sample ID	Clean Reads	Mapped Reads	Mapping Rate (%)	CG Mapped Reads	CHG Mapped Reads	CHH Mapped Reads	CG Coverage (%)	CHG Coverage (%)	CHH Coverage (%)
0h	666,666,674	563,214,247	84.48	416,428,781	551,122,463	180,843,473	93.86	93.96	91.19
1day	666,666,674	560,646,455	84.10	417,790,295	546,768,358	202,369,485	93.22	93.14	90.01
3day	666,666,668	547,894,267	82.18	399,966,903	538,767,041	139,585,914	93.82	93.90	90.83
5day	666,666,672	562,193,704	84.33	384,518,765	509,774,923	381,493,463	92.76	92.58	89.01

Note: Mapping results of four whole-genome bisulfite sequencing samples were shown in the table. Mapping Rate (%) = Mapped Reads/Clean Reads.

Supplementary Table S3. DMGs numbers of three types at different stages.

Group	DMGs
0h_vs_1day.mCG	2,943
0h_vs_1day.mCHG	12,918
0h_vs_1day.mCHH	7,773
1day_vs_3day.mCG	3,693
1day_vs_3day.mCHG	14,612
1day_vs_3day.mCHH	10,929
3day_vs_5day.mCG	1,801
3day_vs_5day.mCHG	4,071
3day_vs_5day.mCHH	1,590

Note: The DMGs were genes with DMRs (|Fold Change| ⩾ 2, and the Fisher's exact test P value ⩽ 0.01) between two samples. For example, 0h_vs_1day.mCG represented the mCG type comparison between 1day sample and 0h sample.

Supplementary Table S4. DNA methylation level of CSC marker genes for mCG/mCHG/mCHH.
